# Proprioceptive Neuromuscular Facilitation Kinesio Taping Improves Range of Motion of Ankle Dorsiflexion and Balance Ability in Chronic Stroke Patients

**DOI:** 10.3390/healthcare9111426

**Published:** 2021-10-22

**Authors:** Donghwan Park, Youngsook Bae

**Affiliations:** 1 Department of Physical Therapy, College of Health Sciences, Kyungnam University, Changwon 51767, Korea; donghwan80@kyungnam.ac.kr; 2 Department of Physical Therapy, College of Health Science, Gachon University, Incheon 21936, Korea

**Keywords:** ankle movement, balance, kinesiology taping, proprioceptive neuromuscular facilitation, stroke

## Abstract

This study aimed to determine the effect of a proprioceptive neuromuscular facilitation (PNF) pattern Kinesio taping (KT) application on the ankle dorsiflexion range of motion (DF-ROM) and balance ability in patients with chronic stroke. This crossover study included 18 patients with stroke. The subjects were randomly assigned to three interventions: barefoot, ankle KT (A-KT), and PNF-KT. The A-KT was applied to the gastrocnemius and tibialis anterior (TA) muscles, and subtalar eversion. The PNF-KT was applied on the extensor hallucis, extensor digitorum, and TA muscles. DR-ROM was measured using the iSen™, a wearable sensor. Balance ability was assessed based on static balance, measured by the Biodex Balance System (BBS), and dynamic balance, measured by the timed up and go (TUG) test and dynamic gait index (DGI). Compared with the barefoot and A-KT interventions, PNF-KT showed significant improvements in the ankle DF-ROM and BBS scores, TUG, and DGI. PNF-KT, for functional muscle synergy, improved the ankle DF-ROM and balance ability in patients with chronic stroke. Therefore, the application of PNF-KT may be a feasible therapeutic method for improving ankle movement and balance in patients with chronic stroke. Additional research is recommended to identify the long-term effects of the PNF-KT.

## 1. Introduction

Lower limb somatosensory impairments are present in a majority of chronic stroke survivors and differ widely across modalities. Chronic stroke is referred to as at least six months after an initial stroke event. The global prevalence of stroke in 2019 was 101.5 million people, and it is the second leading cause of death worldwide, with 50% of the cases leading to chronic movement disabilities due to residual hemiparesis in the limbs [1]. In chronic movement disability, deficits of foot and ankle proprioception are most highly associated with falls [2]. In addition, the spastic foot, which is common in subjects after stroke, is characterized by “foot drop”, which is defined as the inability to raise the front part of ankle and toe, and is due to plantarflexion stiffness, dorsiflexion weakness, and a decreased ankle dorsiflexion range of motion (DF-ROM) [3]. Patients with stroke and decreased ankle DF-ROM have tibialis anterior muscle paralysis, weakness, and/or stiffness [4]. Additionally, bodyweight leaning towards the non-paretic side reduces their balance [5], leading to an asymmetric weight distribution, inefficient gait compensations, and increased incidence of falls [6]. This further reduces postural control [7]. Foot drop can be managed with physical therapy interventions such as ankle-foot orthosis (AFO) [8] and functional electrical stimulation [9,10]. In particular, AFO is commonly prescribed to assist people with stroke to facilitate their ankle-foot function [11]; however, the use of AFO has a disadvantage, in that it can negatively affect walking ability by causing abnormal muscle activity, pain, fatigue, and limited ankle movement [12,13]. Recently, kinesio taping (KT) has long been used as an adjunct during the rehabilitation program in various diseases to strengthen weakened muscles, control muscle tones, improve active range of motion, balance, functional use and gait ability [14,15,16]. According to recent studies, the application of KT can reduce the hyperactivity of the gastrocnemius and increase the activity of the tibialis anterior (TA) in the correction of foot drop (such as neutralizing the foot), and aid in the correction of equinus deformity [17,18,19], with a more positive effect on joint angle and walking ability than AFO in stroke patients with foot drop [20,21]. Other advantages of KT include its low cost and easy application [22]. KT is primarily applied to the anterior tibial muscle groups, which refers to the ankle dorsi-flexor and extensor muscles of the big toe and toes in front of the tibia [18,19,23]. KT is easy to apply compared to other treatment methods; exercise combined with KT may be used to improve the pain and postural balance of patients with back pain [24]. Due to the effect of improving balance and gait [15,18,19], KT is a method mainly used for lower extremity rehabilitation of post-stroke patients [19].

In stroke survivors, gastrocnemius tension is increased, anterior tibial muscle groups are weakened, and the ankle is inverted, resulting in impaired walking. For these reasons, the application of ankle KT (A-KT) [16] and proprioceptive neuromuscular facilitation pattern KT (PNF-KT) was carried out for clinical rehabilitation of patients with stroke [23,25]. The A-KT is used to correct the deformity of the foot in stroke patients, and KT is applied by gastrocnemius inhibition, ankle dorsiflexion facilitation, and eversion function correction, which has improved the patients’ walking ability [16]. The PNF-KT is an intervention used to induce functional muscle synergy and improve functional movement. It is widely used in patients with neurological and musculoskeletal disorders. Therefore, PNF-KT is applied to promote functional movement by facilitating weakened anterior tibial muscles, such as the TA, hallucis extensor, and finger extensor muscles, which improves walking ability [25]. The application of A-KT and PNF-KT has been shown to effectively improve gait in patients with stroke. However, the effect of A-KT and PNF-KT on ankle movement in patients with stroke remains unclear. Furthermore, ankle DF-ROM affects balance ability. 

As such, interventions that are aimed at enhancing gait, and rehabilitation strategies that increase the contribution of the paretic limb in controlling balance while standing, may increase the symmetry of walking post-stroke [26]. Therefore, this study aimed to assess the effects of lower leg PNF-KT and A-KT on the ankle DF-ROM and balance ability of patients with stroke. We hypothesized that the PNF-KT method, which facilitates the anterior tibial muscle group’s function, would provide a better enhancement of ankle DF-ROM in stroke patients with foot drop for balance improvement compared to A-KT, which corrects the ankle. 

## 2. Materials and Methods

### 2.1. Study Design

This study was a three-intervention, randomized, crossover trial, conducted from August 2020 to October 2020. The study comprised three interventions: barefoot, A-KT, and PNF-KT. A washout period was given for 10 min to minimize the learning effect on taping between each condition. All participants were randomly assigned to one of the three sequences (barefoot, A-KT, and PNF-KT; barefoot, PNF-KT, and A-KT; or A-KT, PNF-KT, and barefoot) [27]. Randomization was performed by an investigator not related to the participants. Before the experimental process, the participants were informed in detail about the study procedure and safety, and signed a written informed consent. All procedures were approved by Gachon University’s Institutional Review Board (clinical trial registration number: KCT0005524). We adhered to the Declaration of Helsinki’s ethical principles for medical research involving human subjects.

### 2.2. Participants

A total of 18 post-stroke patients from the rehabilitation center (Kyong-in Hospital, Incheon, Korea) volunteered to participate in this study. The inclusion criteria were as follows: (1) diagnosed with hemiplegia due to hemorrhagic or ischemic stroke for more than 6 months; (2) able to walk independently for over 10 m without assistive devices; (3) no ankle joint damage; (4) a Korean Mini-Mental State Examination (K-MMSE) score above 24 [28]; and (5) insufficient ankle dorsiflexion during the swing phase of the gait cycle. The exclusion criteria were as follows: (1) limbs affected bilaterally and (2) a premorbid or current orthopedic problem involving the lower extremities and spine that could affect balance. 

The number of patients with chronic stroke was determined by performing a pilot test of six volunteers. A power analysis was performed using G-power software (G-power software 3.1.2; Franz Faul, University of Kiel, Kiel, Germany) to achieve a significance α level of 0.05, power of 0.80, and effect size of 0.96 (calculated with a partial η^2^ of 0.48). The power analysis results showed that this study required 13 patients with chronic stroke. At the beginning of this study, 32 patients with chronic stroke were screened. Of these, nine were excluded for not meeting the inclusion criteria, and five did not follow the experimental protocol, resulting in the participation of 18 patients. 

### 2.3. Outcome Measurements

Baseline data, including sex, age, weight, height, time from stroke onset, stroke type, hemiplegic side, and K-MMSE score, were collected for each patient. The primary outcome measure was the ankle DF-ROM, and the secondary outcomes were static and dynamic balance. 

#### 2.3.1. Ankle DF-ROM 

Ankle DF-ROM was measured as active ROM during the timed up and go (TUG) test. The TUG test records the time taken to stand from a chair (50 cm in height), walk 3 m, turn around at an obstacle, walk back to the chair, and sit down. An iSen™ device (STT System, San Sebastian, Spain), consisting of the Sen™ 3.07 software and the STT-IWS WiFi inertial sensor, was used. The iSen is a validated and suitable device used for monitoring and analyzing human movement [29]. It uses wearable sensors and was proposed for use as a multi-purpose data acquisition device for data analysis and to measure patients’ body movements [30]. The sensor recorded and automatically quantified movement to the reference sensor. In this study, we used iSen’s lower training ankle analysis protocol, which utilizes two sensors and collects kinematic data. Sensors include gyroscopes, accelerometers, and magnetometers. Based on the protocol, one sensor was placed on the midline of the affected lateral ankle and another on the tibial tuberosity [31,32]. iSen is conveniently tuned for human motion tracking and the minimum measuring angle is 0.01 degrees. In this study, the DF-ROM was measured using a static roll angle to measure the Y-axis, and the measurement error was less than 0.5 degrees, with the minimal detectable angle being 0.01 degrees [31]. Data were collected at 25 Hz. Data analysis was performed offline. The raw angular data of the dorsiflexion movements were exported to an Excel file, and the maximum and minimum values were obtained. The test was repeated three times and the average value was determined. 

The reliability of the ankle DF-ROM was assessed by randomly selecting six subjects from the participants. These values were measured three times, and the intra-class correlation coefficients (ICC), with a 95% confidence interval (CI), were calculated. The test–retest reliability (intra-class correlation coefficients) for the ankle DF-ROM was 0.904 (95% CI: 0.744–0.964) in this study.

#### 2.3.2. Balance Ability

Static balance was measured using the Biodex Balance System (BBS) (Shirley, NY, USA). Dynamic balance was measured using the TUG test and dynamic gait index (DGI). BBS is interfaced with a devoted software (Biodex, Version 1.08, Biodex, Inc., Shirley, NY, USA). The movable balance platform provides up to 20° of surface tilt in a 360° ROM that enables the device to serve as an objective assessment of balance. The static balance test result includes an overall stability index score, which was acquired from the BBS. The overall stability index was measured as the angular excursion of a patient’s center of gravity. Fluctuations around the zero point, established prior to the testing, when the platform was stable, are presented as the findings of this test. The BBS calculates the overall stability index from the degrees of tilt around the anterior-posterior (sagittal plane) and medial-lateral (frontal plane) axis at a rate of 20 Hz. It separately calculates the medial-lateral stability index and anterior-posterior stability index, whereby Y = the tilt angle of the tilt board in the medial-lateral direction, and X = the tilt angle of the tilt board in the anterior-posterior direction. 

The BBS software measured the angular excursion of a patient’s center of gravity (COG) on the balance board, and calculated a participant’s balance ability with the anterior/posterior index, medial/lateral index, and overall stability index during a given task. These indices were calculated using the degree of oscillation of the platform, and low values showed that the subjects had a good posture stability. The average of three tests was considered the subject index. The overall stability score was a good indicator of the overall ability of the patient to balance on the platform [33]. The system had various difficulty levels for the assessment of balance and fall risk that ranged from 1 (most difficult) to 12 (the easiest). Based on the results of the previous studies, level 12 was chosen as appropriate for all the test participants to perform well [34]. All the tests were performed barefoot and conducted with the eyes open. During a bilateral stance, the participants were instructed to step on the BBS platform and assume a position of comfort; their arms were placed across their hips, and they were asked to maintain a fixed position during perturbation of the rotating plate. The test was performed for 30 s, repeated three times, and the mean value was used. The reliability of the overall stability index of the BBS is 0.92 [35].

The TUG test and DGI were reliable in measuring balance ability in patients with 175 chronic stroke [36]. The DGI was developed to examine the ability of patients to maintain functional balance during the performance of activities during gait [37]. The DGI consists of eight tasks: walking on flat ground, changing walking speed, turning your head left and right while walking, moving your head up and down while walking, turning the axis while walking, crossing obstacles, walking around obstacles, and going up and down the stairs. The DGI has a scale of 0 to 3 for each task, with “0” being the lowest level of function and “3” being the highest, for a total of 24 points. A score of 19 or less is interpreted as a fall risk, and a score of 22 or more is considered safe [38]. A total of three measurements were performed, and the average value was used.

### 2.4. Interventions and Procedures

Each patient performed the tests under the three interventions (barefoot, A-KT, and PNF-KT) in a random order to avoid bias through learning or fatigue. An experienced physical therapist who performed the tests was blinded to the patient intervention assignments and study hypotheses, to avoid expectation bias. Patient characteristics and outcomes (ankle DF-ROM, static balance, TUG, DGI) were assessed by the same physical therapist. A taping was applied by one qualified physical therapist with more than five years of experience who was blinded for the purpose of the study. After each tape application, the patients walked at a comfortable speed on a treadmill for 10 min to familiarize themselves with the three interventions until a proper motion in walking was achieved. Data were collected after walking on a treadmill immediately after the tape was removed. The measurement was repeated three times to obtain an average value. A 10-min rest period was provided between interventions [39]. During application, the tape applied that was not stretched for 5 cm from the initial site was then stretched to 30% for the remaining parts [40].

#### 2.4.1. A-KT

**Figure 1:**
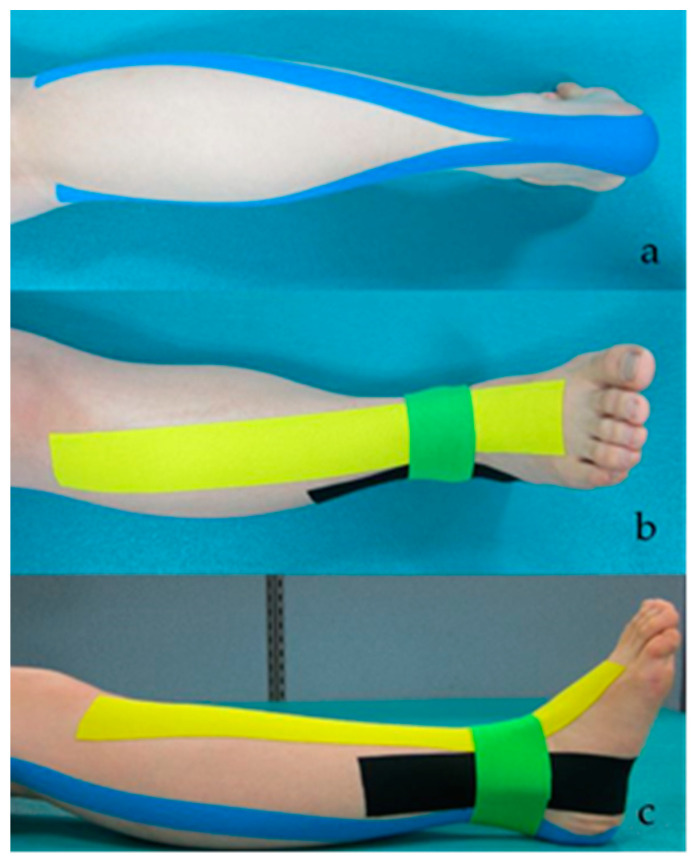
A-KT applied to the gastrocnemius muscle (**a**), tibialis anterior (**b**) and for subtalar eversion (**c**).

For the A-KT, a standard 5 cm 3NS TEX (TS CO., LTD, Seoul, Korea) was used. Muscles are facilitated when the Kinesio tape is applied from the origin to the insertion of the muscle, and are inhibited when applied from the insertion to the origin [41]. The A-KT is used to correct deformity of the foot, and was applied to reduce hyperactivity of the gastrocnemius, increase activity of the TA, and correct eversion. The first taping was applied from the insertion to the origin of the gastrocnemius to inhibit muscle activity in a prone position with an extended knee. Because the subjects’ ankle positions had different ranges of dorsiflexion, the researcher carried out the dorsal flexion manually and applied the tape in a position without pain or discomfort. The second taping was applied from the origin to the insertion of the TA muscle to facilitate muscle activity in a supine position [16]. The third and fourth tapings were attached for functional correction of a subtalar eversion in a supine position, and the tape was placed at a position perpendicular to the midline of the second and third toes, and the midline between the medial and lateral malleoli [16]. The maximum length of the tape was applied from the medial malleolus to the sole to about 10 cm above the lateral malleolus. For the fourth taping, the maximum length of the tape was applied from the medial malleolus to the lateral side of the ankle [16,42] (Figure 1).

#### 2.4.2. PNF Taping

**Figure 2:**
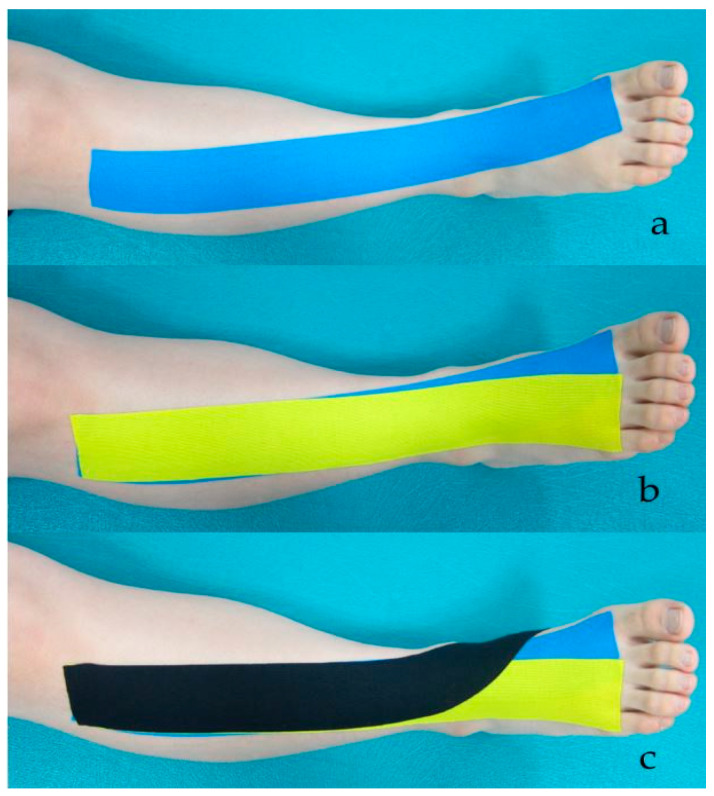
PNF-KT applied over the extensor hallucis muscle (**a**), extensor digitorum muscle (**b**), and tibialis anterior muscle (**c**).

The PNF taping used the maximum length of a standard 5 cm 3NS TEX (TS CO., LTD, Seoul, Korea) tape (Figure 2). The PNF-KT is applied to promote functional movement by facilitating the weakened TA, hallucis extensor, and finger extensor muscles to induce functional muscle synergy. From the extended position of the flexion–adduction–external rotation pattern of the leg, the PNF taping was attached from the origin to the insertion points to facilitate muscle activity of the TA, extensor hallucis, and digitorum muscles in a supine position [25].

### 2.5. Data Analysis

All data were analyzed using PASW Statistics for Windows, version 20.0 (SPSS Inc., Chicago, IL, USA). One-sample Kolmogorov–Smirnov Z-tests were conducted to confirm the assumption of normal distribution. A one-way, repeated-measures analysis of variance was used to assess the statistical significance of the ankle DF-ROM, static balance, TUG test, and DGI among the three interventions. Statistical significance was set at 0.05. Furthermore, the difference between barefoot, A-KT, and PNF-KT was calculated as a delta percentage. The new significance level was 0.05/(comparison number) based on the Bonferroni correction. Therefore, in this study, the adjusted significance level was 0.017 (with α = 0.05/3 = 0.017). All variables are expressed as mean ± SD.

## 3. Results

The average age of the 18 participants was 60.61 years, and the general demographics are summarized in Table 1.

Table 2 presents the test results. There were significant differences among the three conditions for ankle DF-PROM (F = 82.87, *p* < 0.001), static balance (F = 26.75, *p* < 0.001), TUG (F = 15.78, *p* < 0.001), dynamic gait index (F = 33.14, *p* < 0.001). The ankle DF-ROM (*p* < 0.001), BBS (*p* < 0.001), TUG (*p* < 0.001), and DGI (*p* < 0.001) were significantly improved in the PNF taping, compared with those in the barefoot and ankle taping.

## 4. Discussion

This study showed that A-KT and PNF-KT significantly increased the ankle DF-ROM, BBS, DGI, and decreased TUG time compared with the barefoot intervention. These results proved that KT improves the ankle DF-ROM and balance ability of patients with stroke. In addition, PNF-KT enhances ankle DF-ROM and balance ability more than A-KT. These findings confirm our research hypothesis.

Balance disorders can be due to several causes, such as reduced muscle strength and range of motion [9,43]. The recovery of weakened muscles in paretic legs can improve the balance of patients with stroke [42,44]. When muscle strength is recovered, the range of motion is also improved, which manifests in the ankle dorsiflexor strength and static balance [45]. In this study, A-KT and PNF-KT were applied to the TA muscle. KT enhances muscle activation and re-education by increasing the subcutaneous space and providing tactile stimulation and motor recovery [46]. The application of KT on the TA improves balance [47]. Our findings show that the ankle DF-ROM was 7.69° with no taping, 8.62° with A-KT, and 9.62° with PNF-KT. Although both A-KT and PNF-KT showed an ankle DF-ROM of > 8°, the ankle DF-ROM increased by 0.93° in A-KT and 1.91° in KT, and was significantly higher in PNF-KT than A-KT. These results suggest that PNF-KT could be more efficient in walking for stroke patients. However, in the swing phase during walking, as well as ankle dorsiflexion, knee and hip flexion are important factors. Therefore, additional research on this is needed.

In a previous study, ankle correction taping was effective for static balance [48]. In our results, PNF-KT had a 24% higher increase in ankle DF-ROM and a 38% higher decrease in the BBS score than A-KT. Therefore, the authors predict that PNF-KT enhances the synergy of the functional muscles of the TA muscles, thereby enhancing the static balance. In addition, deficits of foot and ankle proprioception are most strongly associated with impaired balance [2]. In this same line, a previous study analyzed the joint position sense (JPS) which means the ability to recognize joint position, and evaluated the proprioceptive function described by the sensory perception of joint position [49]. JPS was significantly improved in the paralyzed ankle after KT [50]. In this study, the authors suggest that additional studies are needed to confirm the change in JPS in order to prove the improvement of static balance after PNF-KT.

Walking ability, such as walking speed, is mainly influenced by the strength of the ankle dorsiflexors [51]. Increased ankle ROM improved the walking speed in patients with stroke [52]. Therefore, increased ankle DF-ROM may increase walking ability. The results of this study showed that the increase in ankle DF-ROM and improvement in the TUG was significantly higher in PNF-KT than A-KT. TUG is closely related to balance ability [53], and a decrease in TUG time means an improvement in functional ability [54]. In this study, compared to barefoot, the TUG time was decreased by 5.1% and 12.1% in A-KT and PNF-KT, respectively. In addition, the DGI score was increased by 19.38% in PNF-KT compared to barefoot. The DGI was designed to evaluate dynamic balance during walking [37]. These results show that PNF-KT application enhances ankle DF-ROM, thereby improving dynamic balance during walking. Accordingly, this study provides new information supporting the hypothesis that the application of PNF-KT in clinical practice is effective in improving ankle DF-ROM and balance ability in patients with stroke. These results suggest that using PNF-KT to enhance the activation of the anterior tibial muscle groups is more efficient than A-KT for functional correction.

This study has some limitations. First, a washout period of 10 min was taken to eliminate the learning effect, but this may not be enough. In addition, the intervention periods in this study were relatively short; therefore, further studies are needed to evaluate the lasting outcome of the intervention, the duration for which the intervention maintains its therapeutic effect, and the economic effects of the therapy. Second, this study is a crossover trial and did not include follow-up tests. Therefore, it is necessary to conduct a randomized controlled trial to confirm the effect of A-KT and PNF-KT. Therefore, the long-term effects of PNF-KT should be evaluated. Third, outcomes were measured using an observational scale. Follow-up studies are needed to determine the changes in walking ability. Lastly, findings may not be generalized for all patients with stroke. The patients in our study had mild or moderate (Brunnstrom motor recovery stage 4 or 5) physical impairment [55]. Additional studies are required to study these specific issues. 

Despite these limitations, this study has several advantages. This is the first study to investigate the effect of PNF-KT for functional muscle synergy on the ankle DF-ROM and balance ability in patients with chronic stroke. Therefore, it provides the basis for further research on PNF-KT for improvement in lower extremity function. Hence, this study has clinical significance because it verifies the improvement of ankle movement and balance through taping.

## 5. Conclusions

This study showed that PNF-KT more significantly improved ankle DF-ROM and static balance than A-KT, as assessed by the TUG test and DGI. These results imply that PNF-KT is a feasible therapeutic method of improving balance and ankle DF-ROM of chronic stroke patients with foot drop during clinical rehabilitation. Taken together, this study will serve as the basis for the development of novel therapeutic interventions and strategies for patients with chronic stroke.

## Figures and Tables

**Table 1:** Baseline characteristics of the participants (*n* = 18).

Sex (male/female)	8/10
Diagnosis (infarction/hemorrhage)	11/7
Paretic side (left/right)	10/8
Age (years)	60.61 ± 13.11
Height (cm)	165.44 ± 6.17
Weight (kg)	66.80 ± 6.75
MMSE (score)	27.12 ± 2.30
Onset time (months)	11.72 ± 4.02
Range of motion of ankle dorsiflexion (degree)	7.69 ± 1.02
Brunnstrom motor recovery stage (4/5)	5/13

MMSE: mini mental state examination.

**Table 2:** Comparisons of variables before and after intervention.

Variables	Barefoot	Ankle KT (Δ% *)	PNF-KT (Δ% **)	F	*p*
Ankle DF-ROM (°)	7.69 ± 1.02	8.62 ± 1.20 (12.0)	9.60 ± 1.25 (24.8)	82.87	<0.001 ^abc^
Static balance (point)	1.02 ± 0.34	0.77 ± 0.37 (24.5)	0.63 ± 0.34 (38.2)	26.75	<0.001 ^abc^
TUG (s)	20.54 ± 4.86	19.49 ± 4.33 (5.1)	18.04 ± 3.64 (12.1)	15.78	<0.001 ^abc^
Dynamic gait index (point)	16.61 ± 1.42	17.72 ± 16.38 (6.6)	19.83 ± 0.92 (19.3)	33.14	<0.001 ^abc^

Abbreviations: KT, kinesio taping; PNF, proprioceptive neuromuscular facilitation; DF-ROM, dorsiflexion range of motion; TUG, timed up and go test; ^a^ *p* < 0.05 indicate a significant difference between barefoot and ankle KT; ^b^ *p* < 0.05 indicate a significant difference between barefoot and PNF-KT; ^c^ *p* < 0.05 indicate a significant difference between ankle KT and PNF-KT; Values are expressed as mean ± standard deviation; * ([ankle KT-barefoot]/barefoot) * 100 (%); ** ([PNF-KT-barefoot]/barefoot) * 100 (%).

## Data Availability

The data associated with the paper are not publicly available, but are available from the corresponding author on reasonable request.
